# Developing Biopsychosocial Research on Maternal Mental Health in Malawi: Community Perspectives and Concerns

**DOI:** 10.1002/eahr.500095

**Published:** 2021-07-01

**Authors:** Lucinda Manda-Taylor, Eric Umar, Robert C. Stewart, Macdonald Kufankomwe, Genesis Chorwe-Sungani, Owen C. Mwale, Demoubly Kokota, Joyce Nyirenda, Kazione Kulisewa, Martyn Pickersgill

**Affiliations:** Department of Health Systems and Policy in the School of Public Health and Family Medicine in the College of Medicine at the University of Malawi; School of Public Health and Family Medicine in the College of Medicine at the University of Malawi; Division of Psychiatry in the College of Medicine and Veterinary Medicine at the University of Edinburgh; College of Medicine at the University of Malawi; Kamuzu College of Nursing at the University of Malawi; College of Medicine at the University of Malawi; Department of Mental Health at the Faculty of Medicine in the College of Medicine at the University of Malawi; College of Medicine at the University of Malawi; Department of Mental Health at the Faculty of Medicine in the College of Medicine at the University of Malawi; Usher Institute at the Edinburgh Medical School

**Keywords:** human research ethics, maternal mental health, autonomy, consent, community engagement, Malawi

## Abstract

Interest in maternal mental health research is growing around the world. Maternal mental health research studies in Malawi have, for instance, sought to determine and establish the incidence and prevalence of depression and anxiety in pregnant people and the factors that contribute to experiences of these states. This article reports stakeholder perspectives on potential community concerns with biopsychosocial mental health research (which might include collecting blood samples) in Malawi. These perspectives were generated through a town hall event that featured five focus group discussions with various participants. In this article, we reflect on key themes from these discussions, demonstrating the endurance of long-standing concerns and practices around autonomy, consent, and the drawing of blood. We conclude by arguing that, while maternal mental health research conducted in Malawi could benefit Malawian women and children, consultation with community stakeholders is necessary to inform whether and how such research should be conducted.

Interest in maternal mental health research is growing around the world. This interest has been propelled by a concern about ill-health implications for mother-infant pairs.^1^ The perinatal and the postnatal periods are together commonly regarded as a time of particular significance. It can be a time of joy and positive expectations and of stress and difficulties.^2^ It is now well established that symptoms associated with several psychiatric disorders can emerge during pregnancy and the postpartum period, with depression and anxiety being the most common.^3^ An estimated 11.9% of women meet criteria for perinatal depression globally, with a higher prevalence in low- and middle-income countries (LMICs) than in more affluent nations.^4^ Risk factors include HIV,^5^ an unwanted pregnancy,^6^ poverty, marital problems or intimate partner/domestic violence, low social support, and the loss of a child or loved one.^7^ In LMICs, these risk factors are shaped by colonial legacies and contemporary international economic policies and practices that can further disadvantage nations experiencing social and economic turbulence.

Malawi has a considerable burden in both communicable and noncommunicable diseases such as HIV, malaria, tuberculosis, and more recently, cardiovascular disease and diabetes. In recent years, Malawi experienced a downward trend in maternal mortality. It achieved significant success in reducing the country’s maternal mortality ratio as per previous targets defined by the United Nations’ Millennium Development Goals.^8^ Much of the nation’s health intervention and research efforts are now directed toward meeting the United Nations’ Sustainable Development Goals, to ensure healthy lives for people of all ages.^9^ For mental health research, the interest is focused on reducing, by a third, premature mortality from noncommunicable diseases through prevention and treatment and promoting mental health and well-being by 2030. Research in maternal mental health in Malawi is one way of contributing toward addressing the U.N. Sustainable Development Goal target 3.4, which aims to reduce by one-third premature mortality from noncommunicable diseases (NCDs) through prevention and treatment and to promote mental health and well-being.^10^

With regard to maternal mental health research, studies in Malawi have, for instance, sought to determine and establish the incidence and prevalence of depression and anxiety in pregnant people in Malawi and the factors that contribute to these problems. Stewart et al. have previously reported a prevalence of DSM IV major depressive episode of 10.7% among antenatal clinic attendees, and 13.9% among mothers of young children attending a child health clinic in Malawi.^11^ In a qualitative study using focus group discussions in rural Malawi, Stewart et al. also found that mental health problems in the perinatal period are recognized and seen as disabling states that warrant intervention.^12^ In a context of poverty and marked gender inequality, threats such as financial insecurity, HIV, and unsafe childbirth are particularly potent stressors.

Building on this work, the recently launched Generation Malawi study (of which authors LMT, EU, RS and MP are coinvestigators), funded by the United Kingdom’s Medical Research Council, is a study of family, maternal, and childhood mental illness. This is a five-year project that aims to extend understandings of mental (ill) health in families (i.e., mothers, fathers, and wider family relations) and how this relates to early childhood development. The project will, among other research activities, involve the collection of biological samples for subsequent genotyping to contribute to knowledge on mental health genomics. This project requires collaboration between Malawian and international researchers to explore treatment and management options for mental ill health. The project will involve several community and public engagement activities to engage, inform, and include communities in various health research activities.

This article reports stakeholder perspectives on potential community concerns with biopsychosocial mental health research (which might include the collection of blood samples), of which Generation Malawi is an example. These perspectives were generated through a “town hall” event that featured five focus group discussions with various participants (as described below). We reflect here on key themes from these discussions, demonstrating the endurance of long-standing concerns and practices around autonomy, consent, and blood drawing. We conclude that while maternal mental health research conducted in Malawi could benefit Malawian women and children, consultation with community stakeholders is necessary to inform whether and how such research should be conducted. Specific research practices such as the drawing of blood need to be particularly closely considered, given, for example, their potential to raise the alarm in communities and their risk of impacting existing public health initiatives.

## Study Methods

We conducted a town hall-style consultation exercise aimed at producing vital qualitative data about how different stakeholders framed maternal mental health disorders and judged the benefits and concerns associated with biopsychosocial research into these. This exercise was undertaken as one way of introducing our ideas for the aforementioned Generation Malawi project. It involved presenting an overview of maternal mental health disorders developed in English by one author (RS) and delivered in Chichewa by another author (EU), followed by focus group discussions. All the focus group discussions took place in a venue in Blantyre, Malawi’s commercial capital (located in the Southern Region, with a population of around 120,000).^13^ Participants were from urban and peri-urban settings in Blantyre district. They included health care workers (formal and informal), traditional and religious leaders, and a range of other community members (mothers, teachers, farmers, and workers at nongovernmental organizations) (see table 1). Data were collected in May 2019.

Researchers’ engagement of the community is critical, particularly for health research that involves collecting biological samples, given the tremendous symbolic power and meaning of blood, bodily fluids, and tissues.

In total, five focus group discussions took place. These were made up of two groups of community members (n = 11), one group of informal and formal health care providers (n = 6), one group of traditional and religious leaders (n = 8), and one group of health professionals or key informants (n = 5) (see table 1). The University of Malawi’s College of Medicine Research and Ethics Committee granted ethical approval. Written informed consent was obtained from all participants during data collection. Literate participants provided a signature on the consent form, and those unable to do so provided a thumbprint. Participants were reminded that their involvement in the research was voluntary and that withdrawal was permitted at any time and without any personal consequence. Participants were assured that their identifying details would be omitted from transcripts and that, to ensure confidentiality, no personal details would be divulged by the research team.

For the focus group discussions, descriptive vignettes (generated by RS in English and translated into Chichewa by EU) of women experiencing maternal mental health problems were used as a prompt to encourage the participants to reflect on and discuss their conceptualizations of the causes, consequences, treatment, and prevention of maternal mental ill health in Malawi. At the town hall meeting that preceded the focus group discussions, we also shared expert reflections on maternal mental health research conducted mainly within the Western biopsychosocial model (via translation by EU). The proposed Generation Malawi epidemiological study of maternal, child, and family mental health in Malawi was also introduced. This was done to encourage the discussion between individual event participants and between these participants and the research team regarding the health, social, and ethical issues that might be encountered in conducting a study like Generation Malawi. We used this approach to generate qualitative data to understand better what kinds of concerns exist around the biomedical and health agendas discussed at the event.

The focus group discussions were facilitated by coauthors MK, KK, DK, GC, OM, and JN. Each focus group facilitator had a note taker (named in the acknowledgments section below) and followed a discussion schedule developed by LMT, MP, EU, and RS. The discussions were conducted in Chichewa, recorded and transcribed into Chichewa, and then translated into English. The town hall event started at 8 a.m. and finished at noon. The focus group discussions were organized into two parts, with a short break in between; each lasted for one hour.

LMT and MP examined all transcripts. Using their broader knowledge of ethics and social science debates and literature, they independently looked for overarching similarities between the concerns raised by participants across the focus groups. These were then more systematically coded by LMT in Microsoft Word. Data quality precluded in-depth discourse analysis but was sufficient to discern dominant themes. Specifically, within the focus group discussions with community members, traditional and religious leaders, and health providers, the data suggested the endurance of community concerns around the drawing of blood and consent and male involvement in maternal mental health research. These require more attention through dedicated qualitative research. In what follows, we set out reflections based on the salient issues that emerged from the data from the focus group discussions (FGD), focusing mainly on consent issues and blood drawing.

## Involving Men in Maternal Mental Health Research

Male involvement in recruitment was reported as very important for the acceptability of maternal mental health research and for enhancing the consent process. One participant offered suggestions about how to include men: “First, check the situation of the family before informing the husbands about the aim of the study. There is a need to talk with the families first and make them aware of the research to better understand before participating in the study” (Participant 6, Community Members FGD).

Engaging men (specifically husbands), as well as other family members, was presented as crucial. This was to minimize any conflict between husbands and wives that recruiting women was regarded as having the potential to stimulate. “If a woman can take part in the study without explaining it to her husband,” a participant said, “there is a possibility that they can engage in conflict. There is a need for women who want to take part in the study to tell their husbands about the research they wish to take part in. This ensures that the husband or relatives should know and give her approval to take part in the study. Failing to do so, she can bring suspicions, as the husband *might* doubt her on what she is doing with the researchers” (Participant 6, Traditional & Religious Leaders FGD).

Some participants emphasized the need to obtain consent from other family or community members, to avoid misunderstandings and conflict between spouses and family members. A health worker noted, “Consent should also be taken from both partners, including family members. The reason for taking consent from both husband and family members is to avoid misunderstandings that might arise if the woman takes part in the study without informing the husband or family members. So, consent of the husband plus family members needs to be taken” (Participant 1, Health Workers FGD).

## Community Engagement and Hiv-Related Stigma

Community engagement was stated as necessary to avoid stigma and social discrimination, mainly because research like the Generation Malawi project could require drawing blood. One health worker recommended, “Explain to the community that the research is not HIV related. This is to prevent participants from being mocked or from facing unintended stigma because community may think that the participants are part of a research on HIV, especially since the participants will be followed up on for five years” (Participant 2, Health Workers FGD).

In Malawi, all pregnant women are tested for HIV (which remains highly stigmatized) to help prevent mother-to-child-transmission of the virus. If a woman tests positive, it is a significant concern because it can create spousal tensions that include violence. These risks can be augmented within longitudinal research, requiring following up participants, since follow-up may arouse familial suspicion and result in stigma as people may assume that the mother and baby are HIV positive. One participant pointed out, “When you collect blood samples, your aim is to test depression but to them [biopsychosocial mental health research participants], might think that you will test HIV, making people have fear” (Participant 1, Professionals FGD).

Involving traditional and religious leaders was also cited as necessary, particularly to strengthen the acceptability of the study and to minimize the concerns around taking specimens like blood. According to another participant, “The moment you decide that you are taking this issue to the community, I feel they should be heavily involved as you have explained to say that you have identified the target population and how you will reach the target group. Since you will be collecting blood samples, make sure that traditional and religious leaders should know about this even though the blood that you will be taking will be small. Do not overlook this; it might negatively impact your study” (Participant 3, Professionals FGD).

## Concerns around the Perceived Intentions of Researchers

Alongside fears relating to HIV/AIDS exist concerns around taking blood, which have much longer histories in Malawi (and other nations within Africa). As Chiwoza Bandawe has observed, in a country with great poverty, underdevelopment, and unemployment, the fear of bloodsuckers reflects people’s experiences of other difficulties.^14^ He suggests that blood drawing is almost a symbolic representation of their life and hope being drawn out of them.^15^ Consequently, medical practitioners can sometimes be on the receiving end of accusations of witchcraft and vampirism, especially in rural areas.^16^ Our focus group discussions reflected these concerns, with one health worker confirming, “People could think that you are bloodsuckers *(kwabwera opopa magazi)*, which is a common issue in Malawi” (Participant 6, Health Workers FGD).

Someone in a different focus group echoed this claim: “Some people may refuse to take part because they might think that researchers have come to collect blood samples for rituals” (Participant 4, Traditional & Religious Leaders FGD).

Research with pregnant people in sub-Saharan Africa is related to rumors of purposive sterilization campaigns.^17^ Such concerns about the morality and intent of researchers were also highlighted by another key informant, who reflected, “Since in this study, you will be dealing with pregnant women and those who have just delivered, some people may think that you want to be killing young children and removing pregnancies on women to reduce Malawi’s population” (Participant 4, Professionals FGD).

## Discussion and Recommendations

Our data reinforce much of what is known about the social and cultural complexities of consent in research in African countries, which is amplified when there is blood collection.^18^ First, let us reflect on our participants’ recommendations to involve men in maternal mental health research.

African or non-Western women’s frameworks and framing of autonomy are often compared with Western notions of autonomy that seek to empower women to make independent decisions on their lives and health. The literature on gender relations in Africa tends to construct the notion of masculinity as one that limits how women are “allowed” to participate and make autonomous decisions, including in their lives, about their health, and about health-related research.^19^ While not directly refuting this, our data encourage understanding and appreciating that a different conceptualization of autonomy exists in Malawi. As others have argued, Africans tend to place a greater emphasis on familial, communal, and cultural autonomy than on individual autonomy.^20^

Our data also shows that research in communities or community-based research seems feasible and acceptable if there is household and community consultation and approval, mainly if the research involves a pregnant woman. Our findings echo those published by Sullivan and colleagues, which illustrate the gender dynamics (including avoiding conflict and violence) and the balance of power and responsibility (collaboration and teamwork) in the consent process.^21^ Our findings also resonate with the writings of feminists and communitarians who have taken issue with individualistic conceptualizations of autonomy and encouraged its reconceptualization as relational. Accordingly, relationships with family, community, and society play a pivotal role in important decision-making in health care and research. Values such as mutual responsibility, cooperation, respect, and interdependence are viewed as key attributes of relational autonomy. Assent at the household level and community consultation and approval for research involving pregnant women or research that collects biological samples illustrate the depth and breadth of human interdependence. Social surroundings and relationships contribute to developing people’s sense of self, identity, and capacity for self-determination.^22^

The practice of shared decision-making illustrates a social structure that mirrors the *ubuntu* spirit—“I am because we are.” In short, our findings underscore that relational autonomy may determine decisions to participate in research in Malawi. Relational autonomy does not exclude the possibility (or even likelihood) of uneven dynamics of power, especially in gendered dyads or networks that can reproduce patriarchal norms. Nor does it negate the central idea in health care or research that the person whose body is, literally, at stake^23^ should have the final say in what is done to her. While we want to challenge reductionistic framings of women in Malawi as necessarily and quintessentially disempowered, we also refuse to act as apologists for misogyny or coercion. Our point is that our participants’ enjoinments to inform family members (especially husbands) before inviting women to engage in biopsychosocial mental health research need to be read through the lens of relational autonomy and (dis)empowerment.

The data from this scoping study further underscore existing concerns in the literature about taking blood for research purposes. These relate to colonial-era stories of White Europeans stealing Black Africans’ blood, which can be read as a commentary on colonial dominance.^24^ Blood thieves were often described as White people or their collaborators, operating at night using European technology, such as medicines and syringes, to extract people’s blood, which they sold or transformed into other commodities such as medicine.^25^ Today, new accounts of vampires can be read as a comment on life in a world of neoliberal democracy, free markets, and the proliferation of nongovernmental organizations within a context of hunger, poverty, corruption, and disease.^26^ In Malawi, talk of taking blood is also associated with fears of AIDS and the arrival of blood testing for HIV.^27^ HIV-related stigmatization is a key social risk to research participation,^28^ relating to fears about receiving a positive result.^29^

Consequently, any biomedical research that involves the collection of blood must make very clear to participants how much blood will be taken, why it is being taken, who will have access to it, and what will happen to it following the study. Blood in Malawi, as in many other countries, has considerable cultural resonance. Consequently, research consent procedures must maximize participant and community autonomy. Potential participants should feel able and be able to decline to have blood collected without any negative implications for them or their families.

Our data suggest three matters that require careful consideration by biomedical researchers. First, there is some potential that taking blood for research purposes might stigmatize participants; some people who took part in our formative study implied that community members might think that people who have blood drawn for the study must have HIV/AIDS. Second, due to this and broader cultural concerns about the taking of blood for research purposes, the possibility exists that incorporating the collection of blood into a research study will increase the likelihood of community disengagement with the broader study and—maybe even other studies. Third, there is a risk that taking blood for basic biomedical research will interfere with public health initiatives that require blood (such as HIV/AIDS testing), since participants may erroneously conclude that research-related blood collection is being taken for HIV/AIDS testing. Hence, they might conclude that there is no need to have a dedicated test.

Researchers must promote more equitable partnerships with communities to enable them to actively shape and guide any community-based research that is being planned. Researchers must be very transparent with communities during the engagement process by clearly communicating the study’s nature and objectives, including the potential risks and benefits. Scientists must provide realistic reflections about the potential impacts of their research for health in the immediate, near, mid-, and longer terms, to mitigate against the possibility that participants will take part in research due to an expectation of being afforded direct clinical benefits that might be impossible. This involves candid discussion of the nature of the research enterprise itself, as a means of underscoring the often-indirect health and community benefits that participation can bring.^30^ Relatedly, academics also need to actively seek to minimize the likelihood of structural^31^ or cryptic coercion^32^ of potential participants into taking part in public health and biomedical research.

Engagement is critical, particularly for health research that involves collecting biological samples, given the tremendous symbolic power and meaning of blood, bodily fluids, and tissues. Consequently, the investigators of any study that might include collecting blood from community participants need to carefully consider whether the potential scientific and health gains from doing so outweigh the feasible participant and population risks of blood collection outlined above. Consideration should be given to using saliva samples where possible for research purposes, rather than blood samples.

## Study Strengths and Limitations

We conducted the focus group discussions and analyzed our data based on our participants’ roles or status in society and the community. We analyzed these views more broadly to inform future biopsychosocial mental health research (and associated social science and ethics research). Our study’s fundamental limitation is that our recruitment strategy and analysis were not based on age or gender. Further, given sample sizes, we were unable to compare or contrast differences and similarities across groups systematically. Another limitation of note is that this study was conducted only in Blantyre. It involved participants who are not engaged in large-scale, long-term, populationbased studies such as those conducted by the Malawi Epidemiology and Intervention Research Unit (MEIRU) in Karonga District (which is rural) and Lilongwe City (which is urban). The MEIRU has a long record of community engagement and of researching communicable and noncommunicable diseases, including through large-scale collection of biospecimens (including blood). Conducting a town-hall event with those sensitized populations may have revealed less concern about blood sampling for research purposes.^♦^

## Figures and Tables

**Table 1:** Participant Information

*Profession or occupation*	*Traditional authority^1^ (T/A) or district*	*Area*
Clinician	T/A Machinjiri	South Lunzu
Nurse	T/A Machinjiri	South Lunzu
Community member (experiences of maternal mental health disorder)	T/A Machinjiri	Machinjiri
Community member (three-month-old baby)	T/A Machinjiri	South Lunzu
Community member (five months pregnant)	T/A Machinjiri	South Lunzu
Community member (six months pregnant)	T/A Machinjiri	Machinjiri
Teacher	T/A Machinjiri	Nyambadwe
Physician (experiences of maternal mental health disorder)	T/A Machinjiri	Mbayani
Community member	T/A Somba	Manase
Project coordinator	T/A Somba	Blantyre
Project officer	Blantyre	Blantyre
Traditional birth attendant	T/A Kapeni	Lunzu
Community member (experiences of maternal mental health disorder)	T/A Machinjiri	Bangwe
Project officer	T/A Somba	Nkolokosa
Traditional birth attendant	T/A Kapeni	Lunzu
Traditional leader	T/A Kapeni	Namame
Chairperson for care organization	T/A Kapeni	Namame
Sheikh	T/A Kapeni	Limbe
Sheikh	T/A Machinjiri	Blantyre
Villager	T/A Kapeni	Namame
Villager (experiences of maternal mental health disorder)	T/A Kapeni	Namame
Nurse	T/A Somba	Zingwangwa
Clinical officer	T/A Somba	Mpemba
Traditional birth attendant	T/A Somba	Mpemba
HSA	T/A Somba	Mpemba
Traditional leader	T/A Machinjiri	Chilomoni
Farmer	T/A Machinjiri	Chilomoni
Religious leader	T/A Machinjiri	Chilomoni
Pregnant woman	T/A Somba	Zingwangwa
Pregnant woman	T/A Somba	Chilobwe

1 Traditional authorities serve as custodians of communities’ cultural and traditional values.

*Focus group discussion participants* Two groups of community members (n = 11) One group of informal and formal health providers (n = 6) One group of religious and traditional leaders (including traditional birth attendants) (n = 8) One group of professionals or key informants (n = 5)
